# Less Genome, More Gain: Genome Reduction Enhances Transaminase‐Producing *E. coli* in a Scale‐Down Bioreactor

**DOI:** 10.1002/elsc.70080

**Published:** 2026-04-27

**Authors:** Gennaro Avolio, Simon Klaffl, Ralf Takors

**Affiliations:** ^1^ Institute of Biochemical Engineering University of Stuttgart Stuttgart Germany; ^2^ Crop Science Crop Protection Innovation Bioprocess Solutions Bayer AG Leverkusen Germany

**Keywords:** bioprocess scale‐up, bioreactor gradients, genome‐reduced strain, heterologous enzyme production, scale‐down system, transaminase

## Abstract

In large‐scale bioprocesses, mixing limitations and design constraints cause the onset of heterogeneous environments, subjecting the cells to continuously changing external conditions, often reducing their performance compared to laboratory conditions. This study evaluated the performance in producing a heterologous transaminase (TA) of a genome‐reduced *Escherichia coli* strain (RM214) in a STR‐PFR scale‐down system, benchmarking it against a wild‐type strain. Under cycles of glycerol limitation and starvation, combined with oxygen limitation in later process stages, RM214 outperformed the wild‐type strain. Due to its lower maintenance coefficient, RM214 showed a remarkable biomass increase of +53% and a boosted final volumetric activity with a +65% increase. These results were achieved with significantly reduced biomass‐specific substrate uptake rates and respiratory parameters, both crucial for optimizing large‐scale processes. This study underscores the applicability and enhanced robustness of genome‐reduced strains in heterogeneous large‐scale environments.

**Abbreviations**
OD: optical density
PFR: plug flow reactor
STR: stirred tank reactor
TA: transaminase
TCA: tricarboxylic acid

## 1 Introduction

When scaling up a bioprocess from ideal laboratory conditions to industrial‐scale production, microorganisms face a variety of environmental stresses and altered conditions that often reduce performance. Scale‐up effects are commonly grouped into chemical, biological, and physical impacts [1]. At a large scale, limited mixing combined with typical bioreactor setups leads to pronounced concentration and environmental gradients. These can include key factors such as nutrients, pH, oxygen, and temperature [2]. Examples of nutrients and oxygen gradients, which are characterized by zones of high concentrations and zones depleted of nutrients, were reported and studied in the literature. It was shown how these heterogeneities affect cell productivity, lowering the performance and increasing the formation of byproducts such as acetate [3, 4, 5, 6, 7].

**Practical Application**

Genome‐reduced *Escherichia coli* can streamline scale‐up of enzyme manufacture under periodic carbon/oxygen starvation exposure typical of large tanks. In stirred tank reactor‐ plug flow reactor (STR‐PFR) cycling tests, the chassis maintained high heterologous transaminase activity at lower substrate uptake and respiration. Practically, this enables (i) retuning feeds to target a lower average biomass‐specific uptake rate, (ii) reducing aeration/agitation at constant output, or (iii) increasing space‐time yield without added utilities. We recommend adding genome‐reduced strains to strain‐selection panels and validating robustness with cyclic scale‐down before tech transfer, easing tech transfer from lab to plant, and improving cost and sustainability.

Cells must adapt to these heterogeneous environments, where they are repeatedly exposed to fluctuating conditions. This requires frequent up‐ and down‐regulation of stress response systems, including mechanisms like the stringent response, with the consequent increase in the maintenance of the cells [2, 8, 9, 10] (Supporting Information).

In this context, the use of strains in which the responses to repeated external stimuli are reduced or removed may be beneficial, lowering the maintenance demand of the cells and allowing an optimal use of the substrate. These genome‐reduced strains derive from the repeated deletion of irrelevant genes by methods of genetic engineering with the purpose of constructing a functionalized cell for a selected application [9, 11]. Several successful examples of genome‐reduced microbial strains have been reported in the literature. For instance, Hirokawa et al. [12], engineered an *Escherichia coli* strain with a reduced genome that displayed a shorter lag phase and achieved higher optical densities in corn steep liquor medium, indicating improved growth performance. More recently, Cordell et al. [13], demonstrated that a genome‐reduced *E. coli* strain produced greater octanoic acid yields under alternating carbon starvation and limitation conditions compared to the wild‐type strain. Additionally, Sasaki et al. [14], streamlined the genome of *Schizosaccharomyces pombe*, resulting in a modest reduction in glucose uptake rate and an extended lag phase, but notably achieving increased ATP levels and enhanced protein production.

Of great interest is the use of multi‐compartment scale‐down reactors in order to mimic large‐scale conditions, such as gradient formation, on a laboratory scale. This strategy enables researchers to study the effects of heterogeneous environments on microbial cell cultures, providing critical insights into cellular stress responses and supporting the development of more robust strains for industrial applications [15].

We conducted a comparative experiment between two heterologous transaminase (TA) producing strains: the wild‐type *E. coli* W3110 and the genome‐reduced *E. coli* RM214. The latter is a genome‐edited *E. coli* K‐12 derivative lacking the flagellar/chemotaxis modules and selected nonessential functions, which was previously engineered by Ziegler et al. [16]. The deletion targets in RM214 were derived from transcriptomic investigations in the stirred tank reactor (STR)‐plug flow reactor (PFR) scale‐down system, which demonstrated that repeated transient glucose starvation induces futile activation of stress‐response modules associated with a substantial increase in ATP maintenance demand [9].

This strain demonstrated enhanced performance in producing eGFP, used as a model product, due to its lower maintenance coefficient. In this study, we evaluated the strain's effectiveness in producing an industrially relevant TA, using glycerol as an alternative carbon source to glucose. Both strains were cultivated in a fed‐batch fermentation within an STR‐PFR system, designed to impose alternating phases of carbon limitation and carbon starvation. Additionally, in the later stages of the process, an oxygen limitation was introduced in the stress zone, subjecting the cells to a dual‐stress environment. Under these large‐scale mimicked conditions, the genome‐reduced strain exhibited a greater production capacity compared to the wild‐type strain.

## 2 Materials and Methods

### 2.1 Bacterial Strains, Media, and Buffer Solutions

The strains used in this study are listed in Table 1.

**TABLE 1:** Bacterial strains used in this study.

Strain	Genotype	Reference/Source
*Escherichia coli* K‐12 W3110	F−, λ−, mcrA, mcrB, IN(rrnD‐rrnE)1	DSM‐18039
*Escherichia coli* RM214	MG1655 Δfk ΔfiA ΔfiC ΔfgNMABCDEFGHIJKL ΔfiEFGHIJKLMNOPQR ΔfhEABcheZYBRtaptarcheWAmotBA ΔcspD ΔaldA ΔgatABCDR ΔuhpT ΔyeeL ΔfxA	16
*Escherichia coli* K‐12 W3110 pLEV‐TA	F−, λ−, mcrA, mcrB, IN(rrnD‐rrnE)1 [pLEV‐TA] (“wild type” strain, abbrev. WT)	This study
*Escherichia coli* RM214 pLEV‐TA	MG1655 Δfk ΔfiA ΔfiC ΔfgNMABCDEFGHIJKL ΔfiEFGHIJKLMNOPQR ΔfhEABcheZYBRtaptarcheWAmotBA ΔcspD ΔaldA ΔgatABCDR ΔuhpT ΔyeeL ΔfxA [pLEV‐TA]	This study

Although *E. coli* RM214 descends from *E. coli* K‐12 MG1655, the wild‐type reference strain selected for this study was *E. coli* K‐12 W3110, serving as a platform benchmark for the experiments.

2xTY medium was prepared by dissolving in demineralized water 16 g L^−1^ tryptone, 10 g L^−1^ yeast extract, and 5 g L^−1^ NaCl. For the agar plates, 10 g L^−1^ agar–agar was added to the solution and then autoclaved. For antibiotic‐containing plates, the medium was supplemented with 50 µg mL^−1^ kanamycin after autoclavation.

The seed culture medium was prepared by autoclaving 10 g L^−1^ glycerol, 4 g L^−1^ (NH_4_)_2_HPO_4_, and 20 g L^−1^ yeast extract dissolved in demineralized water. The medium for the batch cultivation consisted of 7.7 g L^−1^ glycerol, 4 g L^−1^ (NH_4_)_2_HPO_4_, and 31 g L^−1^ yeast extract. For both media, the (NH_4_)_2_HPO_4_ was dissolved and autoclaved separately, and the components were then mixed to avoid any kind of precipitation over autoclavation. All the media were supplemented with 50 µg mL^−1^ kanamycin, and the induction was obtained by adding 0.1 mM IPTG. These were prepared separately and sterilized by filtration and then added to the media.

Feed for fed‐batch cultivation was composed of 500 g L^−1^ glycerol, 0.1 mM IPTG, and 50 µg mL^−1^ kanamycin.

25 wt.% NH_4_OH and 25 wt.% H_3_PO_4_ solutions were used for the pH titration during the fermentation.

Cell samples were diluted using a sterile 0.9% m V^−1^ NaCl solution, which was obtained by dissolving 9 g of NaCl in demineralized water.

### 2.2 Construction of the Production Strains

The TA‐producing strains were obtained by transforming the plasmid into the two working strains. The TA used in this study was provided by Bayer AG in an expression plasmid, and Bayer is currently developing intellectual property (IP) related to the enzyme. The expression plasmid was introduced via standard heat‐shock transformation on the wild‐type strain *E. coli* W3110 and the genome‐reduced strain *E. coli* RM214 built in a former work of our group [16]. Chromosomal edits were introduced by λ‐Red recombineering using pSIM5 and a tetA–sacB positive/negative selection cassette, following established protocols [17, 18, 19]. The transformed expression vector for use in *E. coli* carried the gene sequence of the structural TA under the control of a lactose/IPTG‐inducible promoter (P_T5_‐lacO). Moreover, the plasmid presented *lacI* sequence and ColE1 ori as the origin of replication. Kanamycin resistance was used as a selection marker.

### 2.3 Bioreactor Setup

The fermentations were carried out in a so‐called scale‐down system. This consisted of a two‐compartment system in which the primary reactor was a STR, and the secondary compartment was a PFR, which was used to mimic carbon and oxygen shortages. The PFR was linked to the STR only at the end of the batch phase, contextually to the start of the fed‐batch phase, and the induction of the TA production.

The primary reactor was a 3 L bioreactor (Bioengineering, Wald, Switzerland) equipped with flow baffles and two six‐blade Rushton‐type impellers. The reactor was equipped with a pH sensor (Mettler Toledo, Columbus, USA) to control pH and a pO_2_ sensor for monitoring dissolved oxygen (DO) tension (PreSens, Regensburg, Germany). In the exhaust gas stream, the concentration of oxygen and carbon dioxide was measured by gas sensors (BlueSens, Herten, Germany). During the fed‐batch phase, the feed was constantly added to the reactor by a peristaltic pump (Watson Marlow, Falmouth, United Kingdom). The feed flow was monitored by a balance recording the weight of the stirred feed flask. The secondary compartment was an in‐house‐built PFR with an inner tube diameter of 20 mm and a total volume of approximately 380 mL. In the PFR, oxygen saturation was monitored via two pO_2_ sensors, one placed close to the inlet and the other one close to the outlet of the PFR. The temperature of the PFR was maintained by insulation material and showed a maximum temperature drop of about 2°C at the outlet of the PFR. A diaphragm metering pump (Sigma/1, ProMinent, Heidelberg, Germany) was used to transfer biosuspension from the stirred tank reactor to the PFR after the connection of the two reactors. For a schematic draw of the system or further details, you may refer to [9, 16].

### 2.4 STR‐PFR System Characterization

The mean residence time (τ), the Bodenstein number (Bo), and the variance (σ^2^) are among the main parameters for the system characterization. In our system, the two factors determining these parameters are the aeration rate of the PFR and the pumping rate used to transfer the biosuspension from the primary reactor to the PFR. The goal of the characterization was then to find proper combinations of these two factors in order to fix the desired residence time, maintaining a fluid plug‐flow behavior. For more details, please refer to [13].

### 2.5 Cryostocks, Seed Train, Batch Phase, and Fed‐Batch‐Stressing Phase

A colony from an agar plate was picked and used to inoculate 20 mL of 2xTY media supplemented with 50 µg mL^−1^ kanamycin. The culture was incubated overnight on an orbital shaker at 200 rpm, 37°C. The following day, 15% glycerol cryo‐stocks were made from the cell culture, flash‐frozen with liquid nitrogen, and stored at −70°C.

The seed train started with 50 µL of the cryo‐stock was directly used to inoculate 5 mL of 2xTY medium. This was incubated on an orbital shaker at 200 rpm, 37°C until the culture reached 0.8–1 OD. Then, 1 mL of cell culture was used to inoculate 200 mL of pre‐inoculum medium contained in a 2 L baffled shaking flask. The flask was incubated overnight at 37°C on an orbital shaker set at 200 rpm. The following day, the seed culture, appropriately diluted with sterile 0.9% m V^−1^ NaCl solution, was used to inoculate the bioreactor, complementing the total volume to 1 L batch medium, with a starting cell concentration of 2.5 OD (0.67 g_cdw_ L^−1^). The temperature was set to 30°C and the batch phase lasted approximately 6 h. During all fermentation stages, pH was set to 7.0 and regulated by the automated addition of 25% NH_4_OH or 25% H_3_PO_4_. The DO tension was always maintained above the value of 20% saturation to 1.6 bar of ambient air. The end of the batch phase was determined by the depletion of carbon sources in the medium. This was also confirmed by the stop or a substantial slowdown of the cell growth, which was indicated by a short DO peak. Furthermore, the absence of glycerol in the medium was confirmed by measuring their concentration using an appropriate kit (reported later).

The fed‐batch phase was started by adding the feed with an exponential feeding profile to install a constant growth rate of µ = 0.05 h^−1^. By adding IPTG (0.1 mM final concentration), the production of the TA was induced.

Together with the start of the fed‐batch phase and the induction, the PFR was connected operating as a carbon starvation stress zone. During the first 19 h of the stressing phase, the DO along the PFR was maintained above 20% (referring to 1 bar of ambient air), we called this phase I. After this, we gave the start to the phase II, which lasted for the last 5 h of the process. In this period, we applied together with the carbon starvation stress an oxygen limitation stress along the PFR. This was applied to mimic what could happen in the late stages of a large‐scale fermentation performed in bioreactors with nonoptimal oxygen transfer rates, in which the oxygen demand of the cell culture exceeds the supply.

### 2.6 Statistical Analysis

Data values were reported as mean ± absolute difference to represent variability around the average measurements across duplicates.

### 2.7 Determination of Optical Density and Biomass Dry Weight

The optical density of fermentation broth appropriately diluted with 0.9% NaCl solution from the primary reactor was measured in triplicate at 600 nm on a spectrophotometer (Amersham Biosciences/GE Healthcare, Amersham, United Kingdom). Sample dilution was properly done to meet the linear range of the photometer used (0.1 < OD < 0.3). For measurement of biomass dry weight, quadruplicates of 10 mL broth were centrifuged in weighted glass tubes at 4000 g and 4°C for 15 min. The supernatant was discarded, and the pellet was washed by resuspending in 10 mL of 0.9% m V^−1^ NaCl solution. The suspension was centrifuged again, and the supernatant was discarded. The resulting pellet was dried at 105°C overnight, and glass tubes containing dried pellets were weighed again for the cell dry weight concentration determination. Correlating the OD measured with the cell dry weight, it was possible to determine a correlation factor to convert OD into g_cdw_. This was repeated two times for each strain, using different OD values (low and high values). The correlation factors for the two strains were found to be identical, 0.27 g_cdw_ L^−1^ OD^−1^ for both *E. coli* W3110 and *E. coli* RM214.

### 2.8 Determination of Glycerol and Acetic Acid Concentrations in Fermentation Supernatant

2 mL of cell culture was sampled and centrifuged for 5 min at 8000 rpm, 4°C. The supernatant was then filtered via a syringe filter (0.22 µm) and stored at −70°C until analysis. The analysis was performed using appropriate kits. Glycerol concentration was determined by Glycerol UV Test Kit (R‐Biopharm, Darmstadt, Germany), and acetic acid concentration by Acetic acid UV‐Test Kit (R‐Biopharm, Darmstadt, Germany).

### 2.9 Enzymatic Assay and Determination of Enzymatic Units

The heterologous TA production of the two strains was evaluated by measuring the enzymatic units (U, µmol min^−1^) over process time. This was done by incubating an appropriately diluted cell culture sample from the reactor in a reaction mixture prepared for the enzymatic assay. The reaction mixture was composed of 50 mM phosphate buffer at pH 8.0, 1 mM pyridoxal phosphate, 4 mM acetophenone, and 20 mM isopropylamine. Cells were diluted to OD 10, and then 20, 10, and 5 µL were added to three different reaction mixtures per sample, in order to identify the linear range for the assay. The reaction mixtures complemented with the cells were then incubated for 18 h at 40°C on a thermoshaker set at 200 rpm. At the end of the assay, the reaction was stopped by adding a reaction stopper solution composed of acetonitrile + 1.5% HCl. The whole reaction was conducted in Eppendorf tubes with a final volume of 200 µL. TA activity was assessed using intact, metabolically inactive whole cells, such that de novo transcriptional or translational regulation does not contribute to the measured activity. At the end of the reaction time, the tubes were centrifuged at 10.000 rpm in a refrigerated centrifuge at 4°C. The supernatant obtained from each reaction tube was then analyzed via HPLC to determine the product 1‐phenylethylamine concentration, which was used to calculate the enzymatic units content.

The biomass‐specific enzyme production rate was calculated considering the variation of enzymatic units per milligram of biomass during a given observation time. No enzymatic activity was detected before the induction, so the enzymatic activity before the induction was set to zero.

### 2.10 HPLC Analysis

The supernatant obtained from the enzymatic assay was analyzed via HPLC (Agilent 1100 series) equipped with a diode array detector (DAD) set at 210 nm. The HPLC was equipped with a RP‐C18 phase column (Phenomenex Prodigy 3 µm ODS‐3 100 × 4.6 mm) and the gradient (0 min 95% B; 10 min 30% B; 11 min 5% B; 11.2 min 95% B; 12 min 95% B) was obtained at a flow rate of 1 mL min^−1^ with buffer A (acetronitrile + 0.25 mL L^−1^ trifluoroacetic acid) and buffer B (HPLC H_2_O + 0.25 mL L^−1^ trifluoroacetic acid).

### 2.11 Determination of the Respiratory Parameters

Online data from the off‐gas analyzer was obtained via a BlueSense gas monitor. The oxygen uptake rate (*Q*_O2_, mmol_O2_ L^−1^ h^−1^) and the carbon dioxide emission rate (*Q*_CO2_, mmol_CO2_ L^−1^ h^−1^) were calculated from the off‐gas data using the following equations:

(1) $$Q_{O2}=\frac{V_{G}\cdot p}{V_{L}\cdot R\cdot T}?(y_{O_{2}}^{\mathrm{IN}}-\frac{\left(1-y_{O_{2}}^{\mathrm{IN}}-y_{CO_{2}}^{\mathrm{IN}}\right)}{\left(1-y_{O_{2}}^{\mathrm{OUT}}-y_{CO_{2}}^{\mathrm{OUT}}\right)}\cdot y_{O_{2}}^{\mathrm{OUT}}$$

(2) $$Q_{CO_{2}}=\frac{V_{G}\cdot p}{V_{L}\cdot R\cdot T}?(y_{CO_{2}}^{\mathrm{OUT}}-\frac{\left(1-y_{O_{2}}^{\mathrm{IN}}-y_{CO_{2}}^{\mathrm{IN}}\right)}{\left(1-y_{O_{2}}^{\mathrm{OUT}}-y_{CO_{2}}^{\mathrm{OUT}}\right)}\cdot y_{CO_{2}}^{\mathrm{IN}}$$

where *V*_G_ (L min^−1^) represents the flow of gas in, *p* (bar) standard pressure, *V*_L_ (L) the actual reaction volume in the tank, *R* the gas constant (0.08314 bar L mol^−1^ K^−1^), *T* is the standard temperature (K), and *y* the percentage of the gas expressed as a fraction.

In order to determine the biomass‐specific oxygen uptake rate (*Q*_O2_, mmol_O2_ g_cdw_^−1^ h^−1^) and the biomass‐specific carbon dioxide production rate (*Q*_CO2_, mmol_CO2_ g_cdw_^−1^ h^−1^) the values of *Q*_O2_ and *Q*_CO2_ were divided by the biomass concentration.

### 2.12 Analysis of Total Carbon, Inorganic Carbon, and Organic Carbon in the Supernatant

The suspension was appropriately diluted with demineralized water and analyzed. Analysis was performed with a multi N/C 2100 S composition analyzer (Analytik Jena, Jena, Germany) to yield the total concentration of carbon and inorganic carbon in the cell culture supernatant.

### 2.13 Determination of the Maintenance Coefficient (m_S_, g_CS_ g_Ccdw_^−1^ h^−1^) and true biomass‐substrate conversion yield (*Y*_X/S_^true^, g_Ccdw_ g_CS_^−1^)

To gain quantitative insight into the reduced maintenance coefficient of the genome‐reduced strain RM214 compared to the wild‐type strain W3110, an estimation of Pirt's maintenance coefficient and true biomass‐substrate conversion yield was performed. This estimation was based on total carbon analysis of the samples performed using a total carbon and nitrogen analyzer. Total and inorganic carbon were measured directly, and the organic carbon fraction, which was calculated by difference, was used to quantify the carbon effectively consumed by the cells for the determination of *Y*_X/S_. Using these measurements, alongside biomass production data, we determined Pirt's maintenance coefficient by performing a linear regression of *Y*_X/S_^−1^ versus µ^−1^ using *N* = 16 for W3110 regression and *N* = 18 for the RM214 regression. Uncertainty of regression parameters was quantified as 95% confidence intervals (CI) of the slope and intercept using the t‐distribution with N‐2 degrees of freedom. Confidence intervals for *Y*_X/S_^true^ were obtained by inversion of the confidence limits of the intercept. The intercept was not significantly different from zero, resulting in nonfinite CI for *Y*_X/S_^true^, these intervals were not reported. The values from [11] of maintenance coefficients and biomass yields were converted to a carbon basis, assuming a glucose carbon content of 0.40 g_C_ g_Glc_^−1^ and a biomass molar mass of 27.76 g per C‐mol (corresponding to 0.432 g_C_ g_CDW_^−1^) value taken from [20].

## 3 Results

### 3.1 Biomass (g_cdw_ L^−1^) and Acetate Production (q_Ac_, mg_Ac_ g_cdw_^−1^ h^−1^)

**FIGURE 1:**
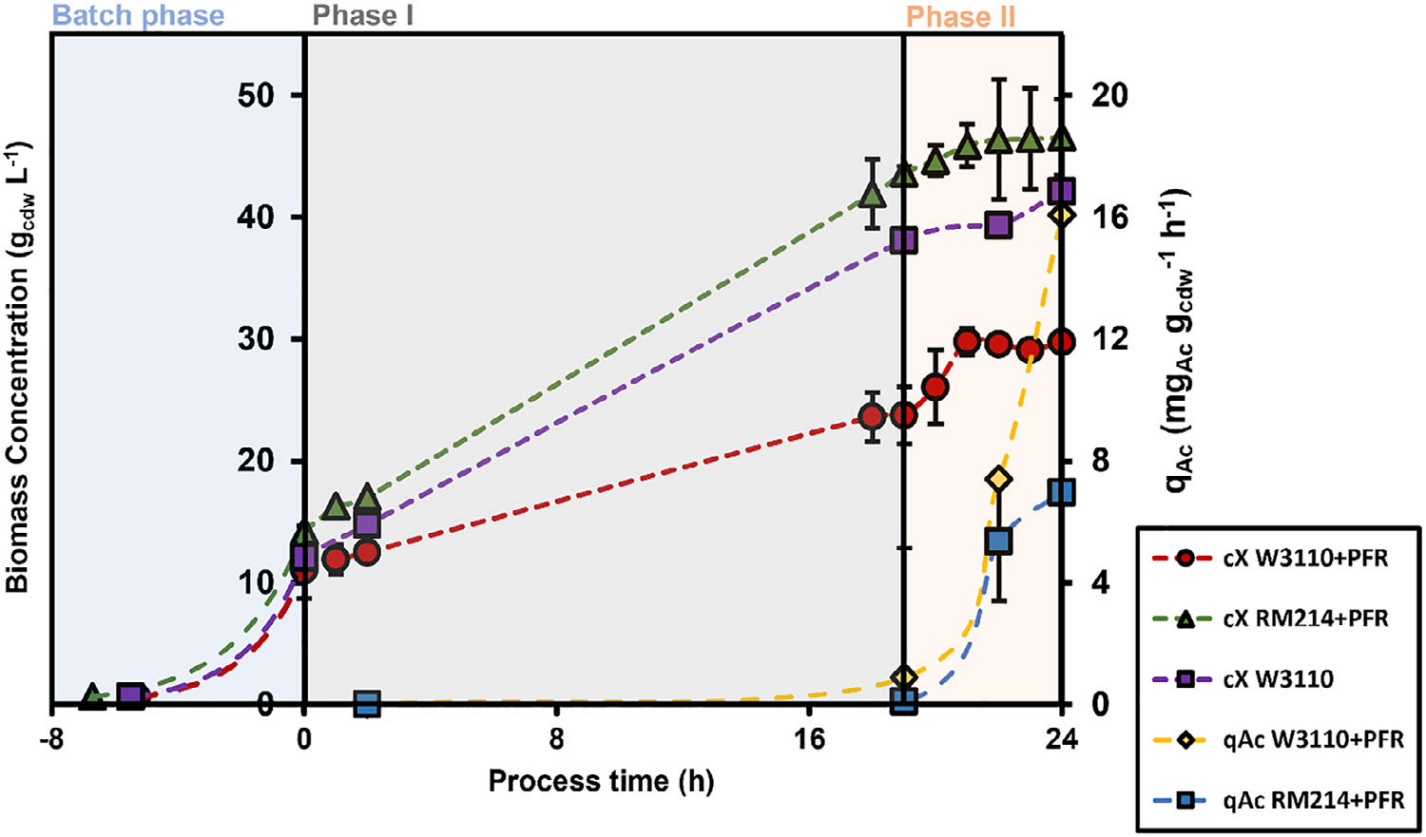
Evolution of the biomass concentration and the biomass‐specific acetate production over the process time. Error bars indicate the absolute difference between the duplicates.

During the batch phase, the maximum specific growth rate (*µ*_max_) was determined to be 0.55 ± 0.03 h^−1^ for *E. coli* W3110 and 0.46 ± 0.01 h^−1^ for *E. coli* RM214. Notably, different biomass concentrations occurred at the end of the batch, which contrasts with the findings of [16] who applied the same strains on glucose. Then, TA synthesis was induced coinciding with the start of the exponential feeding that installed a specific growth rate (*µ*) of 0.05 h^−1^. By linking the PFR to the main STR, the cells were subjected to alternating conditions of carbon limitation and starvation. After 19 h, at the end of phase I, RM214 outperformed the wild‐type strain by achieving 43.65 ± 3.11 g_cdw_ L^−1^ compared to 29.75 ± 0.65 g_cdw_ L^−1^ (Figure 1). The W3110 strain reached significantly higher biomass (42.2 g_cdw_ L^−1^) under nonstressed conditions compared to the stressed scenario. Interestingly enough, the biomass concentration of the nonstressed W3110 was still lower than RM214 under stressed conditions.

Phase II additionally imposed oxygen limitation in the PFR lasting for the final 5 h of the process. As a consequence, the growth rate of RM214 slowed down finally reaching a stationary state, the reference fermentation with W3110 followed the same trend. Additionally, the cells began producing acetate as a byproduct in response to the oxygen limitation in the PFR. The biomass‐specific formation rates of acetate were remarkably higher with the wild‐type than with RM214.

Table 2 reports the amount of glycerol fed over the fed‐batch phase and the ratio of glycerol fed over biomass.

**TABLE 2:** Total glycerol fed and glycerol‐to‐biomass ratio ± absolute error.

Strain	Glycerol fed (g_gly_)	Glycerol fed/biomass (g_gly_ g_cdw_^−1^)
*E. coli* W3110	94.52 ± 7.20	2.68 ± 0.20
*E. coli* RM214	99.66 ± 2.12	1.79 ± 0.13

### 3.2 Enzyme Production, Volumetric Activity (Q_E_, U µL^−1^), and Biomass‐Specific Activity (q_E_, U mg_cdw_^−1^ h^−1^)

**FIGURE 2:**
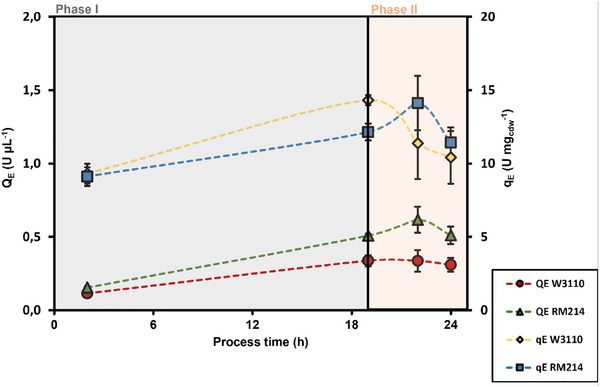
Volumetric activity (*Q*_E_) and biomass‐specific activity (*q*_E_) over the process time. Error bars indicate the absolute difference between the duplicates.

Taking into account the error bars, the biomass‐specific heterologous protein formation rate of both strains was rather similar, revealing a slight rise during phase I followed by weakened production in phase II. However, the volumetric TA production was consistently higher, with RM214 almost doubling the wild‐type performance (Figure 2). In essence, this reflects the likewise increased biomass formation for RM214.

During phase II, the heterologous product formation was more pronounced for the wild‐type strain. (−27%) compared to RM214 (−6%). Likely, enzyme misfolding or aggregation caused the slowed‐down protein production.

**FIGURE 3:**
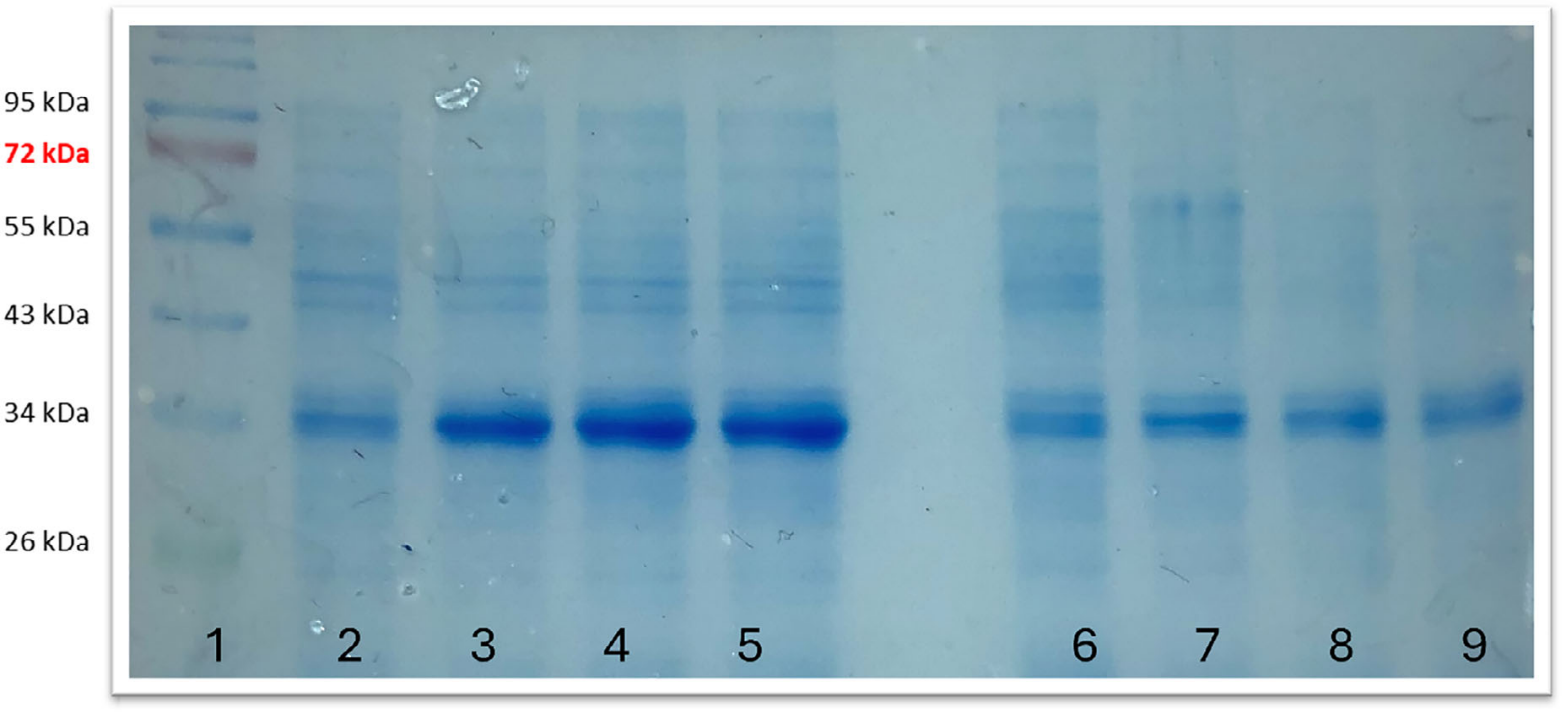
SDS‐PAGE analyses of samples taken during the fermentation.

These findings are further supported by the SDS‐PAGE analysis shown in Figure 3. They confirm that the heterologous TA content per gram of cell was higher in the wild‐type than in RM214 during the late stages of the process. The wild‐type apparently produced more nonactive TA as mirrored by the higher biomass‐specific formation rates of RM214, which are based on TA activity measurements.

Channel 1, prestained protein ladder; Channel 2, *E. coli* W3110, 2 h; Channel 3, *E. coli* W3110, 19 h; Channel 4, *E. coli* W3110, 22 h; Channel 5, *E. coli* W3110, 24 h; Channel 6, *E. coli* RM214, 2 h; Channel 7, *E. coli* RM214, 19 h; Channel 8, *E. coli* RM214, 22 h; Channel 9, *E. coli* RM214, 24 h.

### 3.3 Biomass‐Specific Enzyme Production Rate (qP, U mg_cdw_^−1^ h^−1^) and Biomass‐Specific Substrate Uptake Rate (qS, S g_cdw_^−1^ h^−1^)

**FIGURE 4:**
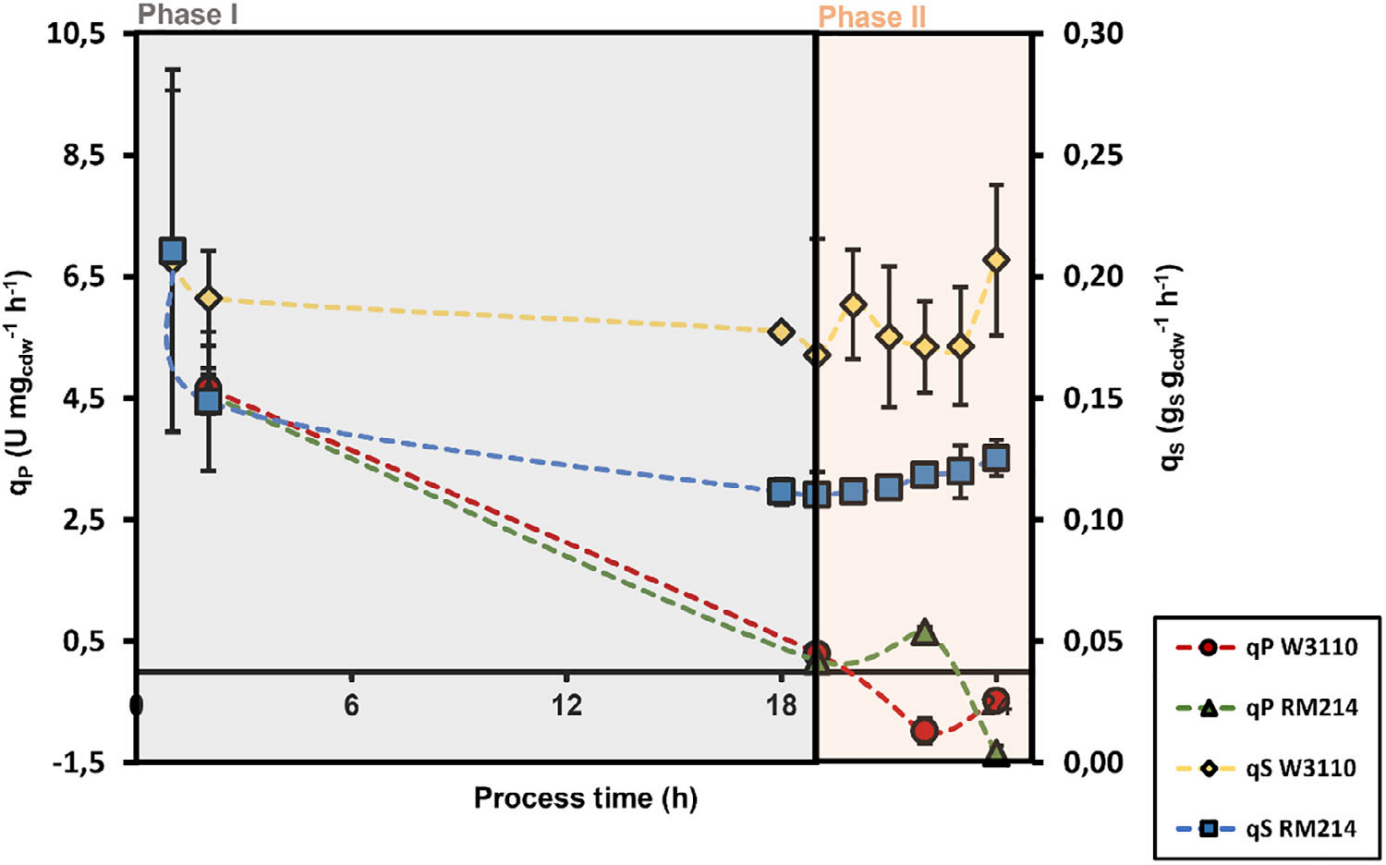
Biomass‐specific substrate uptake rate (*q*S) and enzyme production rate (*q*P) over the process time. Error bars indicate the absolute difference between the duplicates.

As shown in Figure 4, TA production started after induction (time 0). Then, biomass‐specific enzyme production rates peaked before declining until 19 h for both strains. Note that additional measurements between 2 and 19 h are missing. Negative production rates of phase II reflect the start of TA deactivation under oxygen starvation.

Regarding glycerol consumption, the biomass‐specific uptake rate of RM214 was permanently lower than the wild‐type. Nevertheless, RM214 managed to maintain similar product formation rates, which hints at highly efficient carbon‐to‐product flux in this strain.

### 3.4 Respiratory Parameters, Biomass Specific Oxygen Uptake Rate (*q*_O2_, mmol_O2_ g_cdw_^−1^ h^−1^) and Carbon Dioxide Evolution Rate (*q*_CO2_, mmol_CO2_ g_cdw_^−1^ h^−1^)

**FIGURE 5:**
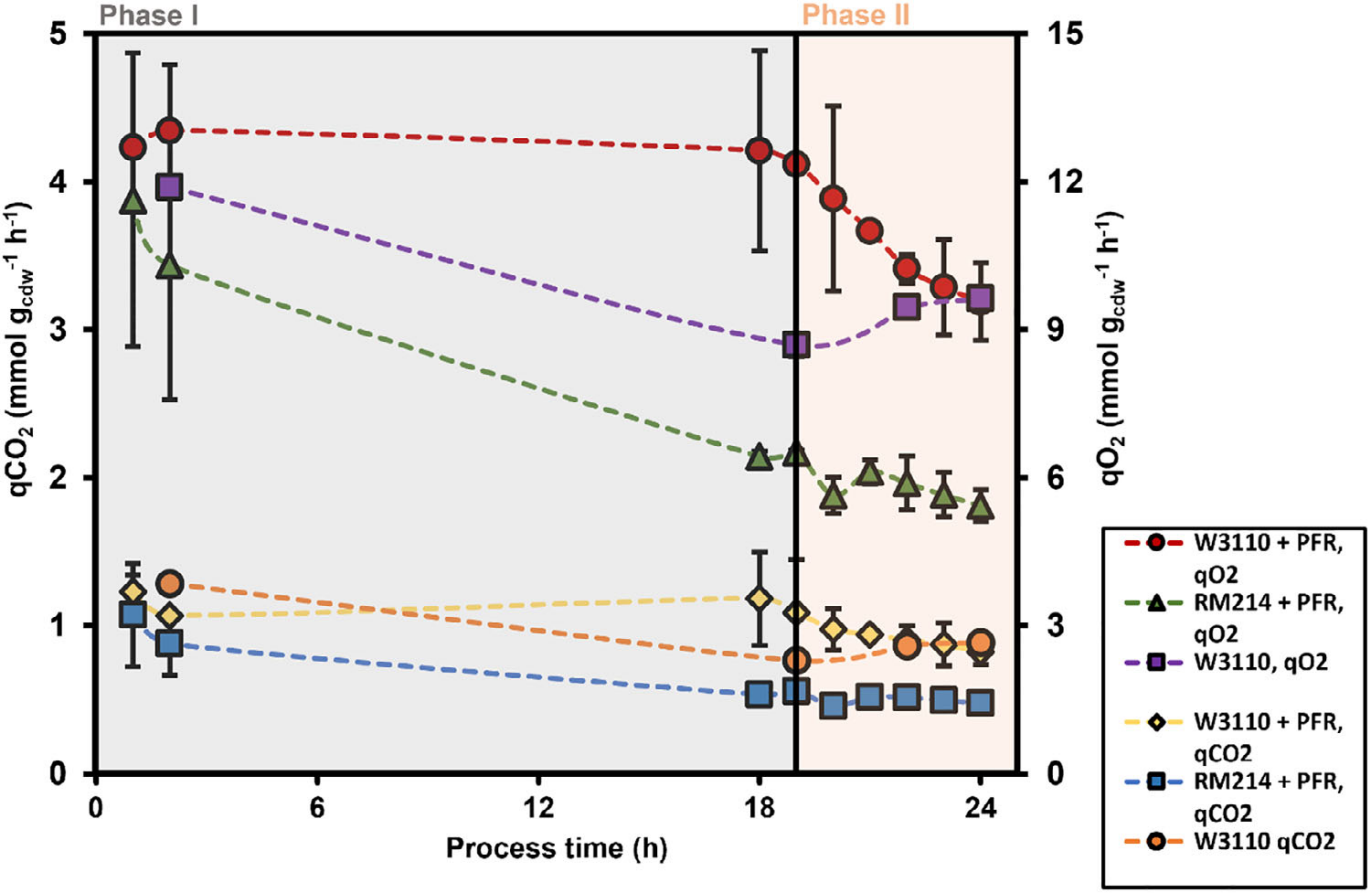
Biomass‐specific oxygen uptake rate and carbon dioxide evolution rate over the process time. Error bars indicate the absolute difference between the duplicates.

The biomass‐specific oxygen uptake rate of RM214 was always lower than related values of the wild‐type strain, irrespective of whether the PFR was connected or not. By analogy, the carbon dioxide evolution rate shows the same trend. These differences are reported in Figure 5. Apparently, the frequent exposure to famine conditions in PFR amplified the need for oxygen uptake in the wild type, which is accompanied by increased CO_2_ release.

The onset of oxygen limitation in phase II limited O_2_ uptake, causing the leveling of oxygen uptake for RM214 and strongly decreasing uptake rates for the wild‐type.

### 3.5 Estimation of the Maintenance Coefficient (m_S_, g_CS_ g_Ccdw_^−1^ h^−1^) and True Biomass‐Substrate Conversion Yield (*Y*_X/S_^true^, g_Ccdw_ g_CS_^−1^)

Further strain comparison was performed by matching the parameters of Pirt's maintenance correlation, that is, *Y*_X/S_^true^ and m_S_.

Ziegler et al. [16] identified the same parameters from steady‐state chemostat cultivations. The current approach used fed‐batch measurements. Nevertheless, the growth‐independent maintenance coefficient m_S_ of W3110 strain was found to be 21% higher than the one of RM214 which is consistent with the 27% difference observed by [16]. In contrast, the values of *Y*_X/S_^true^ observed in this study were significantly higher than those reported in the previous work. Notably, the former study used glucose, whereas glycerol was the carbon source in the current experiments. The detailed results are presented in Table 3.

**TABLE 3:** Comparison of the maintenance coefficient and the true biomass‐substrate yield, as estimated by [16] and in this study ± the estimated interval of confidence (95%).

	m_S_ (this work), g_CS_ g_Ccdw_^−1^ h^−1^	m_S_ [16], g_CS_ g_Ccdw_^−1^ h^−1^	*Y*_X/S_^true^ (this work), g_Ccdw_ g_CS_^−1^	*Y*_X/S_^true^ [16], g_Ccdw_ g_CS_^−1^
W3110	0.116 ± 0.013	0.129	0.65	0.49
RM214	0.092 ± 0.022	0.094	0.94	0.44

## 4 Discussion

### 4.1 Biomass and Acetate Production

Irrespective of whether batch or fed‐batch conditions were applied, RM214 always showed superior biomass production compared to W3110, particularly under stressed conditions with a 53% improvement. This mirrors RM214's property to compensate stress likely due to its streamlined genome, which reduces the starvation‐induced stress regulatory response [16]. Interestingly, the higher *Y*_X/S_^true^ value of RM214 even shows that improved biomass formation is not only limited to stress conditions but also happens during nonstressed growth. This phenotype may be amplified on glycerol, which is a relatively “poor carbon source” for *E. coli*, because it can induce energy‐intensive functions such as flagellar motility [21, 22]. Consequently, the flagella‐deficient strain RM214 can redirect energy from motility to growth, yielding a measurable advantage over the wild type. The flagellar system requires sizeable ATP and proton motive force to carry out its function, as well as the energy required to synthesize it [23, 24]. Thus, the strain likely saves the proton motive force that would otherwise be used to power these systems (estimated to be around 5% of the whole‐cell energy budget [25], as happens in wild‐type strains, resulting in a net advantage reflected in its reduced maintenance coefficient.

The byproduct formation of acetate was lower for RM214 than for W3110, particularly during phase II. This can be attributed to the more efficient management of acetate uptake by RM214 when the cells return to the STR and have access to oxygen again. The process of acetate uptake and its conversion to acetyl‐CoA requires one ATP molecule [26]. Therefore, ATP savings because of reduced maintenance may boost the re‐uptake of acetate by RM214.

### 4.2 Heterologous TA Production

The results of the TA production and activity assays revealed a trade‐off between biomass production and enzyme synthesis in RM214. Despite producing higher biomass, RM214 demonstrated comparable biomass‐specific enzyme activity to W3110 by the end of phase I, which may hint to a limit of maximum heterologous protein production that was already achieved in W3110 and that could not be excelled in RM214. However, the volumetric enzyme activity of RM214 benefits from greater biomass formation, increasing the final performance by about 65%.

These behaviors suggest that the genome‐reduced strain RM214, when exposed to alternating conditions of carbon limitation and carbon starvation, might channel its surplus ATP more toward biomass formation rather than the production of heterologous TA molecules.

The additional stress of oxygen limitation during phase II was better managed by RM214 than by W3110. Given that biomass‐specific values were similar during non‐oxygen‐limited growth, the stronger performance loss of W3110 may reflect increasing cellular demands during dual stress conditions that are smoothed in RM214. The reduction in TA activity, may suggest possible enzyme misfolding or aggregation under stress, especially in W3110, rather than enzyme degradation. The relative surplus of available ATP in RM214 might better maintain ATP‐dependent chaperone activity [27], thereby preventing misfolding.

### 4.3 Substrate Uptake and Respiratory Parameters

RM214 showed lower biomass‐specific substrate uptake rates than W3110, indicating that less substrate is required to enable even improved growth. By analogy, reduced biomass‐specific oxygen uptake and carbon dioxide evolution rates of RM214 anticipate that less substrate oxidation is needed to fulfill energy demands. This is also reflected in the likewise reduced carbon dioxide emission that mirrors TCA cycle activity. Interesting enough, *q*_O2_ and *q*_CO2_ rates of stressed RM214 were even lower than those of nonstressed W3110, the latter being lower than those of stressed W3110. The findings suggest that the alternating exposure to carbon limitation and starvation increases maintenance demands in W3110, for example, by activating ATP‐demanding stringent response and chemotaxis see [16] which require elevated TCA cycle activity. RM214 benefits from lacking those upregulated genes, consequently conserving ATP, reducing respiratory activity, and increasing biomass formation.

The steepest decline of *q*_O2_ observed for W3110 entering phase II further supports this understanding: Acetate formation is known to inhibit TCA cycle activity [28]. Consequently, acetate inhibition is anticipated to impact TCA cycle activity more in W3110 than in RM214.

### 4.4 Maintenance Coefficient and True Biomass‐Substrate Conversion Yield

In the study conducted by [16], the Pirt's maintenance coefficient for the *E. coli* RM214 strain was compared to that of a wild‐type strain. This comparison was made by establishing a steady state through chemostat cultivation and testing various growth rates. The maintenance coefficient and true biomass‐substrate conversion yield were determined by performing a linear regression of *Y*_X/S_^−1^ versus µ^−1^. In contrast, our study made use of a fed‐batch process with exponential feeding at a fixed specific growth rate. Therefore, our investigation focused on only a single growth rate.

In order to compare our results with previous studies and to accurately calculate conversion yields in our fed‐batch process using complex media for the batch phase, we applied a total carbon analyzer for quantifying substrate consumption. This approach allowed us to express both substrate and biomass in terms of grams of carbon, facilitating meaningful comparisons with earlier studies that used glucose as the carbon source.

The results indicate that the W3110 strain exhibits higher maintenance coefficients in both studies, 21% higher in our work compared to 27% in the previous study. Regarding the true biomass conversion yield, we observed differences between the two studies. Current values are higher than those of the previous work. Moreover [22], found that *Y*_X/S_ for *E. coli* on glycerol is 0.52 g_Ccdw_ g_Cgly_^−1^ (converted from g_cdw_ mmol_Cgly_^−1^), which also contrasts with our finding. This may be attributed to the use of complex media in the batch phase for our study, which includes nutrients like amino acids that could facilitate more efficient incorporation into biomass, as opposed to synthesizing them from glucose or glycerol.

The reduced maintenance demand and improved performance observed for *E. coli* RM214 under dynamic cultivation conditions are consistent with the transcriptomics‐guided strain design framework proposed by [9]. In that work, repeated transient starvation in STR‐PFR systems was shown to induce large‐scale oscillations of stress‐response and process‐irrelevant regulatory programs, leading to a substantial increase in ATP maintenance requirements. RM214 was subsequently engineered by [16] based on these findings through the targeted removal of selected stress‐related and non‐essential modules. Within this context, the present study does not aim to reestablish the underlying transcriptomic causality, but rather provides a physiological and process‐relevant validation of this design strategy in a heterologous enzyme production scenario.

### 4.5 Limitations and Generalizability

Although RM214 descends from MG1655, we used *E. coli* K‐12 W3110 as the WT strain for the comparison. As part of another independent strain engineering program, the latter was preferred as it is almost isogenic versus MG1655, differing only in a few loci [29]. As pointed out by Hayashi et al. [29] there are only a few structural differences, such as: *dcuA* frameshift causes growth impact on C4 dicarboxylic acids, *gatA* defect causes non‐use of galactitol, changes in *tnyA*/*tnaB* may impact tryptophan utilization. While those structural changes do not interact with the experimental conditions the stop of *rpoS* in W3110 – which is still active in MG1655 – might do. We therefore acknowledge that W3110 does not represent the direct parental strain of RM214, and that differences in global stress regulation associated with the genetic background of the reference strain may influence the quantitative interpretation of maintenance‐related phenotypes.

To explicitly address this aspect, we have added a dedicated Appendix that evaluates the impact of *rpoS* deficiency under STR‐PFR feast‐famine conditions. The Appendix provides independent supporting evidence showing that the absence of *rpoS* results in only minor changes in global transcriptional responses and does not lead to major deviations in key process performance parameters (e.g., yields and rates) (Supporting information S1).

Our data show that RM214 outperforms the wild type under STR‐PFR glucose feast–famine cycles when benchmarked against *E. coli* K‐12 W3110. This mirrors [16] and [13] using *E. coli* K‐12 MG1655, and supports that the superior performance of RM214 is robust across different K‐12 genetic backgrounds, rather than being attributable to a single parental strain or regulatory element alone. Nevertheless, a direct comparison between RM214 and its immediate parental strain MG1655 under identical STR‐PFR conditions would further strengthen the mechanistic attribution of the observed phenotype and represents an important direction for future work.

## 5 Conclusions

Scaling up bioprocesses from the lab to the industrial scale often encounters performance losses due to gradient formation. In this study, we compared the performance of two heterologous TA‐producing strains using a scale‐down system that mimics large‐scale heterogeneities.

The genome‐reduced strain *E. coli* RM214 demonstrated superior biomass production, achieving higher volumetric activity throughout the process while maintaining biomass‐specific activity despite exposure to dual stress conditions. Additionally, it exhibited a lower biomass‐specific substrate uptake rate and reduced respiratory parameters, which are crucial for large‐scale bioreactors with mixing constraints. We want to draw attention to the evidence that the lower biomass‐specific substrate and oxygen uptake rates have many important implications in industrial processes. These parameters impact both the economic side and the feasibility of a correct scale‐up of the process. In the case of the wild‐type strain that converts more substrate into carbon dioxide (which can be considered a non‐valuable byproduct), it will perform a process with a bigger loss of financial resources, compared to RM214, and this makes the process financially less appealing or, in some cases, not financially sustainable at all. Correlated to this, consider that the lower oxygen requirements of the cells are particularly significant when considering large‐scale bioreactors, where oxygen transfer rates are often constrained by limitations in mixing and aeration, reducing the formation of byproducts as acetate.

The findings have significant implications for optimizing industrial bioprocesses, particularly for large‐scale production of technical enzymes. The enhanced performance of this genome‐reduced strain under stress conditions suggests that genome reduction may contribute to improved strain performance in large‐scale fermentations, although the influence of the specific genetic background of the reference strain cannot be fully excluded. This makes *E. coli* RM214 a promising chassis for large‐scale production of heterologous proteins due to its reduced maintenance, opening new engineering opportunities for this strain, whose streamlined genome also suggests its use in producing a broader range of products.

## Author Contributions

G.A., R.T., and S.K. conceived and designed the research. G.A. conducted the experiments, performed the analyses, and carried out the data interpretation. G.A. wrote the manuscript with reviews and feedback from R.T. and S.K. All authors have read and approved the final version of the manuscript.

## Funding

The authors have nothing to report.

## Ethics Statement

This study did not involve human participants or animal experiments and therefore did not require ethical approval.

## Consent

The authors have nothing to report.

## Conflicts of Interest

The authors declare no conflicts of interest.

## Supporting information




**Supporting File:** elsc70080‐sup‐0001‐Appendix1.pdf.

## Data Availability

The data that support the findings of this study are available from the corresponding author upon reasonable request.
