# Supplementary material for: Less Genome, More Gain: Genome Reduction Enhances Transaminase‐Producing E. coli in a Scale‐Down Bioreactor
Source: Eng Life Sci. 2026 Apr 27;26(4):e70080. doi: 10.1002/elsc.70080 (PMC13112000; doi:10.1002/elsc.70080)
Supplement: Supplementary file 1 — Supporting File: elsc70080‐sup‐0001‐Appendix1.pdf. [file ELSC-26-e70080-s001.pdf]

Results and tables are reprinted from: "Strategies to improve scale-up performance based on metabolic and transcriptomic responses of *Escherichia coli* to substrate heterogeneity" Simen, J. (2020), PhD thesis, University of Stuttgart, Germany.

Appendix Note S1

Similarity of transcriptional responses between WT and ΔrpoS under STR–PFR feast/famine conditions

To assess whether differences in *rpoS* status alone lead to major alterations in transcriptional behavior under STR–PFR feast–famine glucose dynamics, we refer to independent STR–PFR experiments comparing *E. coli* LJ110 WT and its isogenic LJ110 Δ*rpoS* mutant (both W3110-derived), as reported in the public PhD thesis of Joana Simen (University of Stuttgart, 2020).

Under short-term glucose starvation in the PFR, the thesis reports a highly similar transcriptional response between WT and Δ*rpoS*, stating that: *"Overall, the correlation between the transcriptional profiles of WT and ΔrpoS mutant cells at P5 was high (r = 0.81) and increased with increasing residence time in the PFR (i.e. from P1 to P5), which suggests that the fast regulatory response to short-term glucose starvation is coordinated and of comparable extent in both strains. The commonly regulated genes reflect the immediate transcriptional response to glucose starvation that appears to be largely unaffected by loss of rpoS."* (Figure 4.32)

For long-term adaptation to glucose fluctuations, the thesis further reports a substantial overlap in transcriptional regulation between both strains, noting that:

"62 genes showed similar long-term transcriptional regulation to glucose fluctuations in ΔrpoS mutant and WT cells, corresponding to 51 % and 47 % of all DEGs, respectively." "Global correlation of the log2FC of WT and ΔrpoS mutant cells was moderate (r = 0.43), indicating the presence of common, but also σS-dependent regulation." (Figure 4.33)

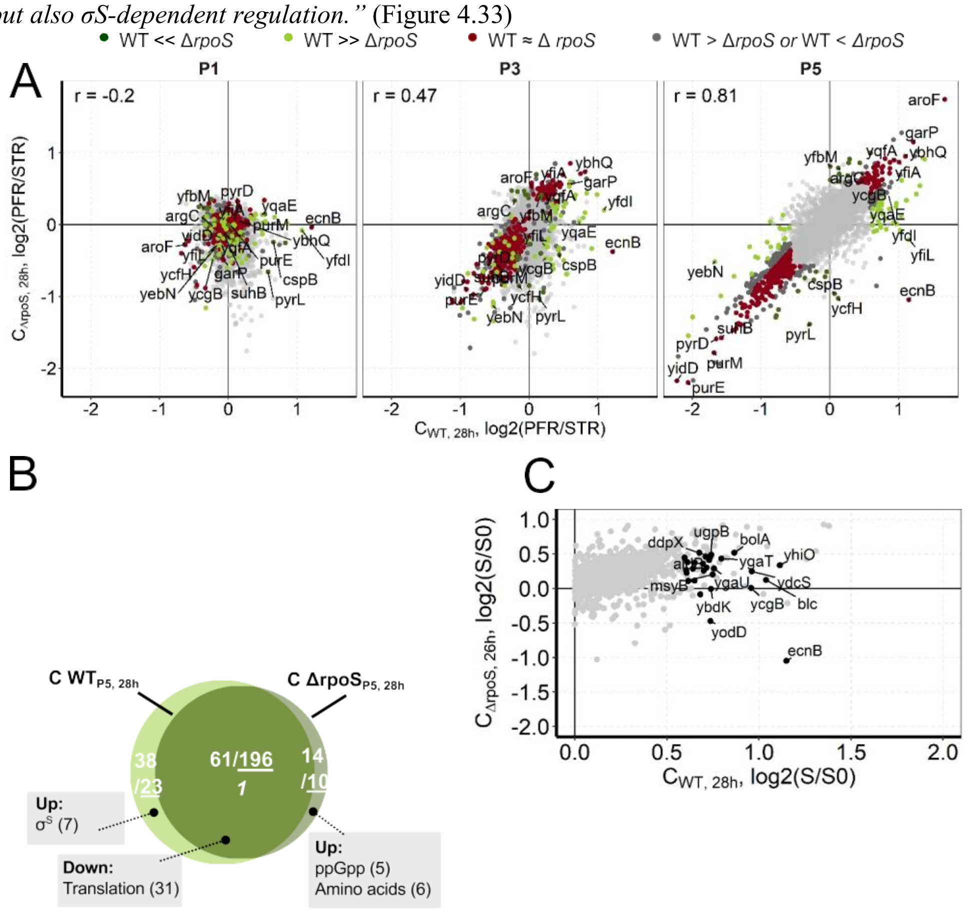

Figure 4.32: Comparison of the short-term response to glucose shortage along the PFR between *E. coli* WT and a Δ*rpoS* mutant. **(A)** Scatter plots of log2FC (P1, P3, and P5 vs. S) of genes sampled after 28 h (n = 3940). Genes are color coded according to the grouping described in Section 3.9. **(B)** Venn diagram representing (overlapping) gene sets for the comparison of P5 vs. S (τ = 110 s). Genes with FDR<0.01 and log2|FC|≥0.58 in at least one dataset are shown. The number of up- and downregulated genes in each set is indicated by regular and underlined numbers, respectively. The number of genes commonly regulated but in opposing directions, is shown in italics. Significantly enriched (FDR<0.05) functional categories are indicated. **(C)** Detail view of **(A)** showing the log2FC of known ppGpp and/or σ^S regulated genes that are significantly upregulated in the WT. r, Pearson's correlation coefficient.

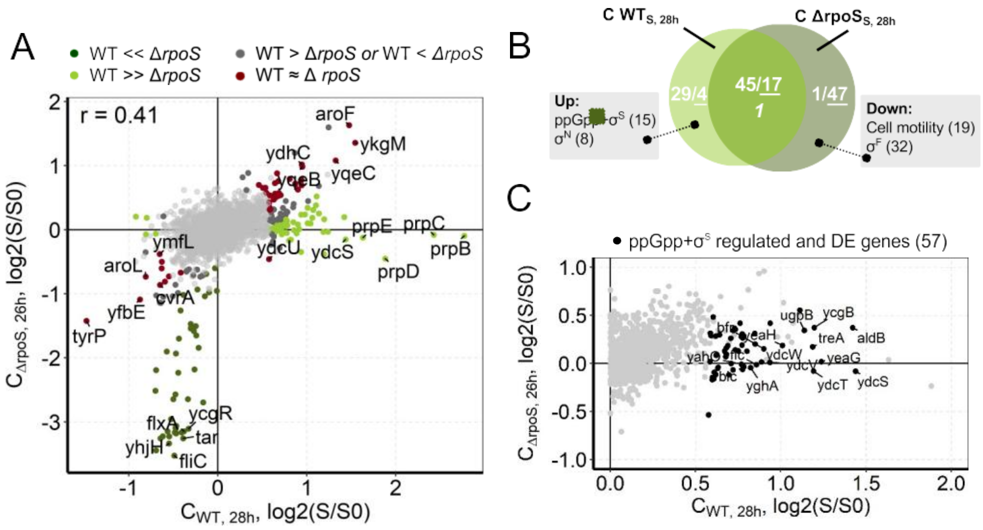

Figure 4.33: Comparison of the long-term response to repeated short-term glucose starvation of *E. coli* WT and the Δ*rpoS* mutant. **(A)** Scatter plots of log2FC (S_1 vs. S_0) for individual genes sampled after 28 h ($n = 3940$). Genes are color coded according to the grouping described in Section 3.9. **(B)** Venn diagram of S_1 vs. S_0 including only genes with FDR<0.01 and log2|FC|≥0.58 in at least one dataset. The number of up- and downregulated genes in each set is indicated by regular and underlined numbers, respectively. The number of genes commonly regulated but in opposing directions, is shown in italics. Significantly enriched functional categories by Fisher test (FDR<0.05) are indicated. **(C)** Detail view of **(A)** showing the log2FC of known ppGpp and/or σ^S regulated genes that are significantly upregulated in the WT. *r*, Pearson's correlation coefficient.

Appendix Note S2

Supporting evidence: minor impact of Δ*rpoS* on process performance under STR-PFR feast/famine conditions

To address potential confounding effects caused by differences in *rpoS* status across K-12 backgrounds, we provide independent supporting data from prior STR-PFR scale-down experiments performed in *E. coli* K-12 comparing a WT (W3110 F nr+) strain to an isogenic Δ*rpoS* mutant. In these experiments, key process parameters were reported to be highly comparable between Δ*rpoS* and WT cultivations at S0 (STR steady-state at time 0 h, before PFR connection), including biomass-glucose yields, specific glucose uptake rates, specific CO_2 production rates, as well as the absence of side-product formation and carbon recovery.

Accordingly, the STR–PFR dataset supports that, under feast–famine glucose dynamics, Δ*rpoS* does not cause major deviations in the main process performance parameters typically used for benchmarking strain performance in bioprocess studies.

The supporting values are provided in Supplementary Table D.4 (WT) and Supplementary Table D.5 (Δ*rpoS*), reprinted from the public PhD thesis of Joana Simen (University of Stuttgart, 2020).

Source statement (verbatim): "Overall, the key process parameters at S0 were highly comparable between Δ*rpoS* and WT cultivations (see Suppl. Table D.5 and Suppl. Table D.4). For example, Δ*rpoS* biomass-glucose yields (S0: 67.0 g_{DW} mol^{-1} (C1) and 65.8 g_{DW} mol^{-1} (C2)) did not significantly deviate from those of the WT (S0: (67.8 ± 1.2) g_{DW} mol^{-1}). Moreover, the specific glucose uptake and carbon dioxide production rates as well as the absence of side product formation were constant between strains at S0. The same is true for carbon recovery."

Table D.4: Summary of key process parameters of glucose-limited STR-PFR cultivations with *E. coli* LJ110 WT. Values are shown for the initial steady-state prior to PFR connection (S0) and for the final steady-state of the STR-PFR system (S1).

	Dilution rate (h^{-1})	$Y_{X/Glc}$ (g/mol)	Glucose medium ($mmol\ L^{-1}$)	Specific rate ($mmol\ g_{DW}^{-1}\ h^{-1}$)			Carbon recovery (%)
				Glucose uptake rate	CO_2 production	Side products	
S0 (STR)	0.2	67.81±1.15	≤0.022	17.70±0.52	9.45±0.25	–	93.56±4.03
S1 (STR-PFR)	0.27/0	73.35±1.73	≤0.022	16.36±0.58	9.03±0.21	–	98.69±4.01

[a] The ammonia concentration in the medium was (87.28 ± 2.42) $mmol\ L^{-1}$ at S0 and (89.26 ± 2.24) $mmol\ L^{-1}$ at S1.

Table D.5: Summary of key process parameters of glucose-limited STR-PFR cultivations with *E. coli* LJ110 Δ*rpoS*. Values are shown for the initial steady-state prior to PFR connection (S0) and for the final steady-state of the STR-PFR system (S1). C1, cultivation 1; C2, cultivation 2.

	Dilution rate (h^{-1})		$Y_{X/Glc}$ ($g\ mol^{-1}$)		Glucose medium ($mmol\ L^{-1}$)		Specific rate ($mmol\ g_{DW}^{-1}\ h^{-1}$)						Carbon recovery (%)	
							Glucose uptake rate		CO_2 production		Side products			
	C1	C2	C1	C2	C1	C2	C1	C2	C1	C2	C1	C2	C1	C2
S0 (STR)	0.2	0.2	66.99	65.77	≤0.02	≤0.02	18.10	17.64	9.48	9.01	–	–	94.62	92.69
S1 (STR-PFR)	0.27/0	0.27/0	74.79	73.45	≤0.02	≤0.02	16.22	17.16	8.19	7.86	–	–	97.69	96.25

[a] The ammonia concentration in the medium was 97.05 $mmol\ L^{-1}$ (C1) and 88.89 $mmol\ L^{-1}$ (C2) at S0 and 88.39 $mmol\ L^{-1}$ (C1) and 81.20 $mmol\ L^{-1}$ (C2) at S1.
